# Dr. Milton Josiah Robert’s Private Home

**Published:** 1893-03

**Authors:** 


DR. MILTON JOSIAH ROBERTS has opened a Private Home, at 122 West 71st Street, New York City, for the special treatment of patients afflicted with deformities, diseases or weaknesses of the spine, as well as joint and bone diseases generally, also other deformities and disabilities of the body. All the comforts of an elegant and quiet home afforded.

